# Evaluation of nursing students’ learning using realistic scenarios with and without debriefing*

**DOI:** 10.1590/1518-8345.2936.3187

**Published:** 2019-10-07

**Authors:** Rita de Cassia Silva Vieira Janicas, Nádia Zanon Narchi

**Affiliations:** 1 Centro Universitário das Américas, São Paulo, SP, Brazil.; 2 Universidade de São Paulo, Escola de Artes, Ciências e Humanidades, São Paulo, SP, Brazil.

**Keywords:** Nursing Education, Learning, Simulation Training, Debriefing, Educational Technology, Patient Simulation, Educação em Enfermagem, Aprendizagem, Treinamento de Simulação, Debriefing, Tecnologia Educacional, Simulação do Paciente, Educación en Enfermería, Aprendizaje, Entrenamiento de Simulación, Debriefing, Tecnología educativa, Simulación del Paciente

## Abstract

**Objective:**

to compare the clinical performance of nursing students in learning scenarios with and without debriefing in a simulation center.

**Method:**

a longitudinal, prospective, interventional, crossover randomized study, with a quantitative approach and before-and-after type, with a population composed of 120 nursing students distributed randomly between experimental and control group. The study phases included theoretical and demonstrative practice on child immunization; first Clinical Performance Test, which served as baseline measurement; randomization; scenarios with debriefing for the experimental group and without debriefing for the control group, according to clinical performance/intervention examination; exchange of groups or crossover; third Clinical Performance Test.

**Results:**

debriefing was proven to be effective in improving the performance of the students in the clinical exams, because improvement in the performance of the experimental group both in relation to the baseline measurement examination and in comparison with the control group in the post-intervention performance examination and in the third examination, after crossover (p<0.001).

**Conclusion:**

the use of scenarios with debriefing constitutes a strategy facilitating the teaching-learning process in the undergraduate nursing course.

## Abstract (pt)

**Objetivo:**

comparar o desempenho clínico de discentes de Enfermagem em cenários de aprendizagem com e sem *debriefing* em um centro de simulação.

**Método:**

estudo longitudinal, prospectivo, de intervenção, randomizado em *crossover*, de abordagem quantitativa, do tipo antes e depois, com população composta por 120 discentes de Enfermagem dispostos aleatoriamente em grupo experimental e de controle. As fases da pesquisa incluíram ministração de aula teórica e prática demonstrativa sobre imunização infantil; primeiro exame de desempenho clínico, que serviu de medida basal; randomização; realização de cenários com *debriefing* pelo grupo experimental e sem *debriefing* pelo grupo-controle; segundo exame de desempenho clínico, de intervenção; troca de grupos ou *crossover*; terceiro exame de desempenho clínico.

**Resultados:**

constatou-se que o *debriefing* foi eficaz para melhorar a atuação dos alunos nos exames de desempenho clínico, pois houve melhora no desempenho do grupo experimental tanto em relação ao exame de medida basal quanto em comparação com o grupo controle no exame de desempenho pós-intervenção e no terceiro exame, após o *crossover* (p <0,001).

**Conclusão:**

o uso de cenários com *debriefing* constitui estratégia facilitadora do processo ensino-aprendizagem na graduação em Enfermagem.

## Abstract (es)

**Objetivo:**

comparar el desempeño clínico de discentes de enfermería en escenarios de aprendizaje con y sin debriefing en un centro de simulación.

**Método:**

estudio longitudinal, prospectivo, de intervención, aleatorio en crossover, de enfoque cuantitativo, del tipo antes y después, con población compuesta por 120 discentes de enfermería dispuestos aleatoriamente en un grupo experimental y en un grupo control. Las etapas de la investigación incluyeron la impartición de clase teórica y práctica demostrativa acerca de la inmunización infantil; primer examen de desempeño clínico, que sirvió de medida basal; aleatorización; realización de escenarios con debriefing por el grupo experimental y sin debriefing por el grupo control; segundo examen de desempeño clínico, de intervención; grupos cruzados o crossover y, por último, tercer examen de desempeño clínico.

**Resultados:**

se constató que el debriefing fue eficaz para mejorar la actuación de los alumnos en los exámenes de desempeño clínico, ya que hubo mejora en el desempeño del grupo experimental tanto respecto al examen de medida basal como en comparación con el grupo control en el examen de desempeño posintervención y en el tercer examen, tras el crossover (p <0,001).

**Conclusión:**

el uso de escenarios con debriefing constituye una estrategia facilitadora del proceso enseñanza-aprendizaje durante el grado de enfermería.

## Introduction

Undergraduate nursing education has experienced challenges arising from the current conceptual and methodological changes occurring in higher education in the health field, which arise in response to the professional profile desired in a increasingly complex, demanding and constantly changing labor market.

From this perspective, the active methodologies are suitable for this demand because they are based on the theoretical principle of autonomy. As they are student-centered, these methodologies encourage students to take an increasingly active stance, which effectively seeks the achievement of learning objectives, in an environment of freedom, support, and protection^(^ 1 ^)^.

Among the strategies of active methodology, one highlights the realistic simulation, which, as a strategy of nursing education, is defined as a technique that uses technologies to replicate scenarios that simulate the practice in a controlled and realistic environment. In it, the student actively participates in the teaching and learning process to practice exhaustively, learn, reflect, and evaluate products and processes^(^ 2 ^)^. When performing scenarios, the student is faced with a reality that will require his/her knowledge for its resolution. This challenge promotes the mobilization and integration of the contents learned, which will be incorporated and will serve as basis for future clinical decisions. Also, it can lead the student to a self-reflection regarding his/her degree of knowledge and/or emotional balance, giving him/her the opportunity to invest in his/her performance and to improve it, through metacognitive thoughts^(^ 3 ^)^.

Considering the importance of metacognition, which is the ability of the human being to monitor and autoregulate cognitive processes^(^ 3 ^)^, great interest from educators in the search for understanding its relationship with the mental functions related to learning is observed.

The clinical reasoning in Nursing is an essential element of professional practice and qualified nursing care, for it is present in the nurses’ actions and decisions. Despite the great importance attached to clinical reasoning, the development of this competence and the transfer of this knowledge to the professional practice is a major challenge for the category^(^ 4 ^-^ 6 ^)^.

Thus, authors^(^ 7 ^)^ report that the ability to solve problems will probably be greater and faster if teaching enables developing cognitive skills that allow the student to become gradually autonomous. When the student is encouraged to develop metacognitive thinking, he/she starts to spot the mechanisms interfering in his/her learning. The use of strategies that will help him to learn more efficiently^(^ 3 ^-^ 7 ^)^ is desirable.

In the simulation, immediately after performing the scenario, the students return to the observation room and join their professors and colleagues to participate in the debriefing, which is a reflection on the conducts performed during the scenario and should occur in a pleasant and reliable atmosphere^(^ 8 ^)^.

During the debriefing, students have the opportunity to explore their emotions, identify their processes of thought, clinical judgment, and nursing conduct^(^ 8 ^)^. In this process, the teachers’ mediation must be planned and include considerations aimed at promoting reflective thinking to help students understand the correlations between patient data, clinical condition, and appropriate nursing conduct^(^ 8 ^-^ 11 ^)^.

These characteristics of simulated teaching have been increasingly valued in the field of vocational training, which makes their inclusion in the nursing teaching-learning process imperative. However, its incorporation has been impaired by issues related to financial resources, sensitization, and teacher’s training. Moreover, its effectiveness needs to be proved, since the investigations in this field still show methodological fragilities, which hinders the production of the gold standard scientific evidences required for generalizations and recommendations.

Thus, considering the need to assess the importance of debriefing in nursing education, the choice was to conduct this investigation to compare the clinical performance of students in learning scenarios with and without debriefing in a simulation center. The hypothesis to be proved is that nursing undergraduate students have a better clinical performance when debriefing is used after the realistic scenarios.

## Method

This is a longitudinal, prospective, interventional, randomized crossover study, with a quantitative approach and before-and-after type, conducted at a high-tech simulation center of a private university located in the East of the municipality of São Paulo and offers several courses in the health field.

Two hundred and twenty students of the fourth semester of the undergraduate nursing course who attended the course *Processo de Cuidar nas Etapas do Ciclo Vital I* (Process of caring in the stages of Vital Cycle I), in which the professional module starts and contents focused on the primary care are addressed, participated in the investigation.

To participate, students should be enrolled, be regularly attending the theoretical classes of the course and should not have previous experience in child immunization, the topic chosen for the conduction of this study. One highlights that all students from the three classes of the fourth semester, two from the morning shift and one from the night shift, met these inclusion criteria, and none of them refused to participate.

**Figure 1:**
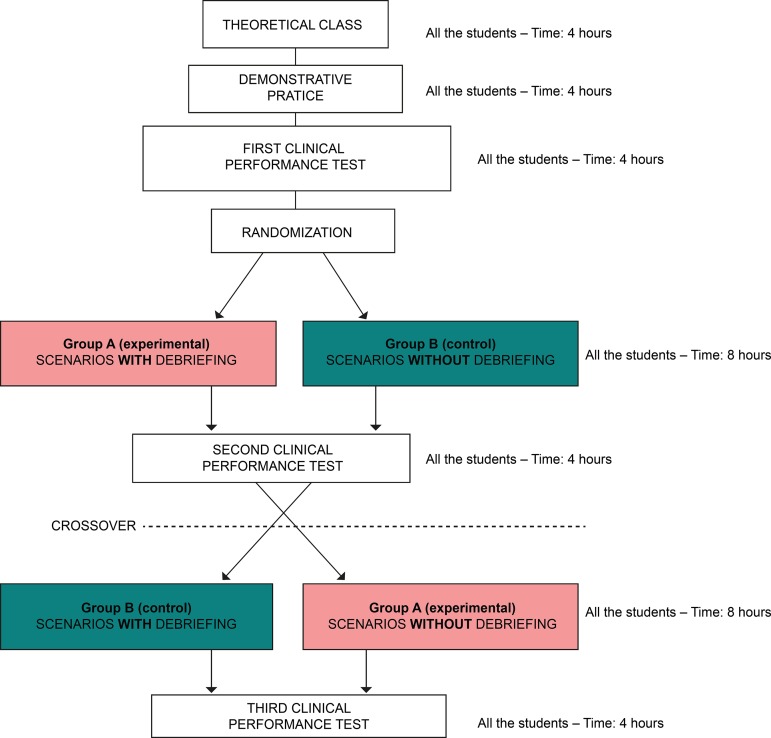
Logistics of the data collection process. São Paulo, SP, Brazil, 2016

Data were collected between August and November 2015 and followed the steps shown in Figure 1.

The simulated classes, in which realistic scenarios with debriefing were used, were considered as *exposure*, and the clinical performance of the students was considered as *outcome*, investigated using the Clinical Performance Test (*Exame de Desempenho Clínico* - EDC).

The course was offered weekly by a teacher who addressed concepts of child immunization, immunization schedule, types of vaccines, and nursing technical procedures applied to vaccination, with the appropriate feedback from the students. The types of classes taught by this teacher were expository dialogue and demonstrative practice. After these classes, the first EDC was done to verify the retention of the contents taught, whose results served as a baseline measurement for randomization, made with statistical support.

The EDC consisted of an assessment of the students’ clinical performance when performing a child immunization scenario. In a contiguous room, separated by a unilateral display, the teacher performed the evaluation based on a checklist with 30 verification items, whose value defined ranged from 0 to 10 points, representing the sequence of nursing actions in the care in the vaccine room. This tool was elaborated by the researchers based on the Manual of Norms and Procedures for Vaccination of the Brazilian Ministry of Health and evaluated in its clarity by two nurses specialized in collective health and with more than five years of experience in vaccination.

One highlights that the strategies were inverted (crossover) to ensure equal opportunities for learning for both groups. Moreover, the teacher who conducted the course did not participate in the stages of data collection or in the correction of the three EDCs.

In the randomization, the terciles of the students’ scores in the aforementioned test were considered the intervening variable to the teaching-learning process to divide the groups into experimental and control. After randomization, the experimental group (A) was composed of 59 students (49.2%); and the control group (B), of 61 students (50.8%).

The three EDCs were administered by nurses with teaching experience who were not teachers at the institution and were duly trained to fill out the checklist. These nurses did not know the students nor the groups to which they belonged, which was a strategy used for blinding the study. The EDC were corrected by one of the researchers who also did not know to which group the students belonged.

The scenarios were performed in simulated rooms equipped with child mannequins and inputs needed for a vaccine room. These scenarios, which lasted up to 10 minutes, were always performed by two students who took the role of the mother of the child to be vaccinated and the nurse who performed the care. This division was made in rotation for all students, so that they could go through both experiences of clients and of health professionals.

The scenarios with and without debriefing were conducted by the teacher of the course, with logistical support of two monitors. The debriefing model^(^ 12 ^)^ used by the teachers of the teaching institution, which is the field of this investigation, follows the following steps: initial phase of welcoming of the students by the debriefer who perform the scenario, aimed at easing the embarrassment and/or reducing the stress to concentrate on the actions performed and not in the participants, with a positive focus on the learning opportunity; phase of synthesis, in which one seeks to homogenize the contents observed by the participants and spectators, and the mediator asks one of the participants to describe his/her experience without value judgment. Other members of the group can complement the description, but without judging the scene, because the data collection will serve as the basis for the discussion - the most important element - at that time; phase of discussion, in which the strengths and the areas for improvement by the group are emphasized, and the facilitator guides the discussion to the learning objectives. The last phase is the summarization of what has been discussed, highlighting potential items for improvement that the student can use to complement and/or to improve his/her studies.

One observed that, in the scenarios without debriefing, the students participating needed to talk about their performance. The doubts arisen from this experience mobilized them to use the theoretical content and the literature related to the topic, once the teacher could not intervene.

The data collected in the three EDCs were tabulated in an electronic spreadsheet, using the software Microsoft Excel^®^. The analysis was performed using the software R 3.2.3. The results are shown in tables of absolute and relative frequency comparing the groups regarding sociodemographic data. The difference in the relative frequencies between groups was assessed by the Fisher’s exact test.

The final scores of the EDC are represented by box plot charts, and by measures of position (average) and scale (standard deviation) statistics. To evaluate the effect of the groups over time, ANOVA models were adjusted for repeated measures, estimated by generalized least squares, and the tests considered a significance level of 5% (p<0.05).

Before data collection, the students were invited to participate in the investigation and oriented about its objectives, as well as about the ethical procedures and the need to sign the informed consent form. This study was approved by the Research Ethics Committee of the School of Nursing of the University of São Paulo (CAAE: 44045115.6.0000.5392) and the participants were duly informed about the current ethical recommendations.

## Results

The characterization of the students showed 109 (90.8%) were female and 11 (9.2%) were male, aged between 19 and 35 years, and the prevalent age group was from 20 to 24 years old (54.1%). Regarding marital status, most of them (87.2%) reported being single. One emphasizes that these characteristics were similar in the groups control and experimental, which provided balance and minimized possible influences or confusion on the effects of the experiment.

By investigating the professional experience, similarities were observed between the two groups. The majority, both in the experimental group (A) and in the control group (B), reported not working (63.3%), while 36.7% reported working in the health field, as auxiliary nurses or nursing technicians in hospitals (14.7%), trainees (10.1%), or in other positions. All students had previous experience with simulation, because they participated in activities at the university’s simulation center since the first semester.

**Figure 2:**
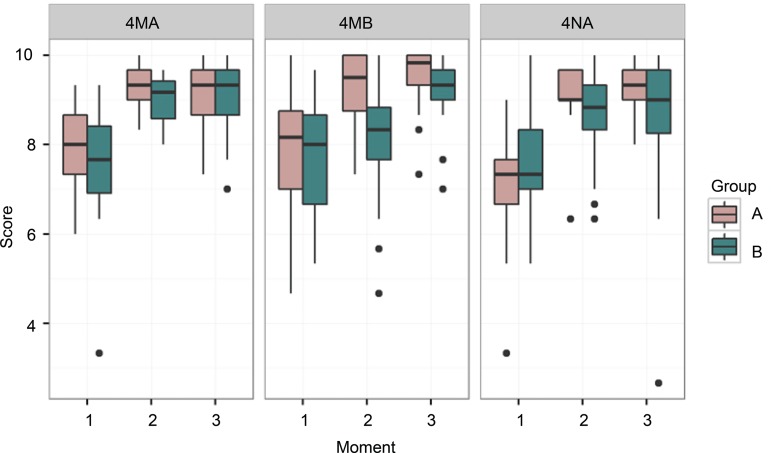
Boxplot of the scores of evaluations per moment, group, and class. São Paulo, SP, Brazil, 2016 (N= 120)

The three EDC scores, expressed in three moments, were represented by box plot charts, and by measures of position (mean) and scale (standard deviation), in the three classes that comprised the study population, that is, in two morning-shift classes (4MA and 4MB) and in the night-shift class (4NA), as shown in Figure 2.

**Figure 3:**
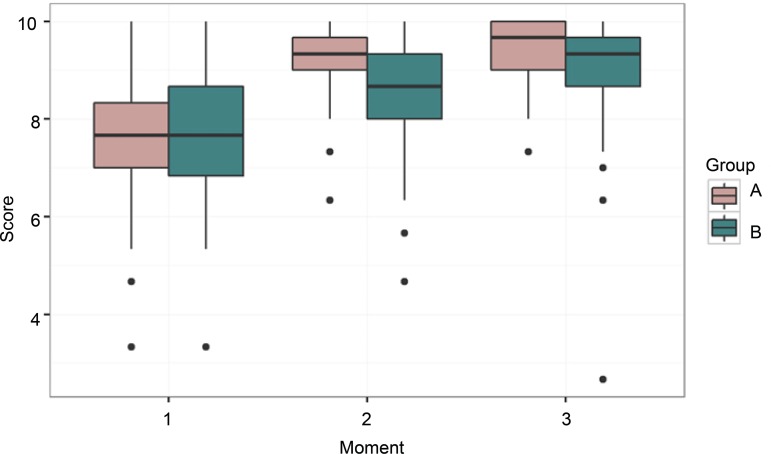
Boxplot of the score per moment and group. São Paulo, SP, Brazil, 2016 (N= 120)

Figure 2 shows group A (experimental) showed better results in the post-intervention phases, that is, in the moments 2 and 3, which correspond to the second and third EDC. Figure 3, which shows the junction of the three classes (morning shift and night shift) and the overall results in the three moments, shows this difference more clearly.

Figure 3 shows the apparent reduction of the group B (control) at moment 2 (according to EDC) is maintained. To test this effect, an ANOVA model was adjusted for repeated measures with interaction between group and time, which analyzes whether the effect of the groups was different over time. This is shown both in Table 1, which shows the averages of the scores represented in Figures 2 and 3, and in Table 2, which shows the estimates of the model coefficients with significant interaction effect (p<0.001).

**Table 1:** Averages and standard deviations of the scores per group in the Clinical Performance Tests. São Paulo, SP, Brazil, 2016 (N= 120)

Moment of evaluation	Average score and standard deviation Group A (n=59)	Average score and standard deviation Group B (n=61)
1 (first test)	7.68 ± 1.22	7.66 ± 1.28
2 (second test)	9.21 ± 0.69	8.44 ± 1.12
3 (third test)	9.37 ± 0.68	8.94 ± 1.18

**Table 2:** Estimated coefficients of the regression model for repeated measures. São Paulo, SP, Brazil, 2016 (N= 120)

Coefficient	Estimated	Standard error	T-value	P-value
Group A (experimental)	7.674	0.140	54.905	<0.001
Second test	1.528	0.165	9.274	<0.001
Third test	1.690	0.164	10.319	<0.001
Group B (control)	-0.046	0.197	-0.233	0.816
Second test	-0.742	0.232	-3.206	0.002
Third test	-0.378	0.230	-1.643	0.101

Table 1 shows the results between the groups before the intervention do not point out that the groups were previously different, strengthening the evidence that the debriefing scenarios were actually effective for learning. At moment 3, this difference decreased again. The Group B showed a slight improvement, equaling to the score of the Group A.

Table 2 shows a significant effect of the second EDC. In other words, the effect of the averages between the groups B and A at moment 2 was 0.742 points lower (p=0.002) in the control group (B).

The distribution of correct answers in each item in the second and third EDC between groups A and B, not shown in tables, showed significant differences in various items of the checklist in the experimental group, which corroborates the assertion that this group had a better performance.

## Discussion

When evaluating the evolution of the groups at the three moments, in general, there was a similar relation of the effect of the group over time, with initial similarity between the two groups after randomization, which can be observed at moment 1 in Figures 2 and 3, strengthening the evidence that debriefing scenarios were really effective for learning.

The average score of all students in the first EDC was 7.6 points (Table 1). One emphasizes that, at that phase, the students had participated only in the theoretical expository dialogue class and in the demonstrative practice; however, even without performing other activities, most of them had a good performance in the EDC of baseline measurement. It is an test of the type “shows how”, classified in the third category of the Miller’s Pyramid^(^ 13 ^)^, in which the student is required to demonstrate knowledge, skills and attitudes to solve the problem situation presented.

This result shows the importance of the theoretical foundation, which has been contributing to provide the theoretical foundation of several teachings for a long time and is one of the most used teaching resources, especially when the intention is to explain concepts, action mechanisms, and treatments, quickly and to many students.

The theoretical class and the practices taught are believed to have aroused the interest from the students, because of the teacher’s method and the topic, which is directly related to the practice of the nursing professional exercise. When associated with potentially significant contents, this willingness of the students to learn is the basis for meaningful learning, which becomes lasting when the teaching strategy instigates the student to seek answers for a better understanding of the content. This results in the learning by discovery, which is very different from that automatic or receptive learning, which shows worse retention^(^ 14 ^-^ 15 ^)^.

The results show the experimental group showed the best learning outcomes, expressed in the scores of the second and third EDC, both in relation to the baseline measurement and in comparison with the control group, which is shown in Figure 2. Table 2, in turn, shows that a statistical significance was observed for the learning of this group that participated in the debriefing.

As mentioned, debriefing is a moment of reflection on the practice developed, in which students explore their actions and emotions and the processes of thoughts that influenced their decision making and develop their ability to evaluate themselves, criticize, and hear criticism, learning from this rich experience^(^ 8 ^,^ 16 ^-^ 18 ^)^. Considering that, debriefing has been identified as one of the most targeted learning opportunities^(^ 17 ^-^ 18 ^)^.

The need for feedback shown by the students of the control group after the traditional classes shows they did not show better results than those of the experimental group, even having sought answers to their doubts. The mobilization of these students to discuss their performances and seek answers in the literature brought some learning benefit, so that they progressed in the second EDC. This need also shows the importance of debriefing as a guide and facilitator of the teaching-learning process.

The role of debriefer has been highly valued as a structural element involved in the debriefing process^(^ 8 ^,^ 17 ^-^ 19 ^)^. As mentioned, the teacher takes a key role in the success of the strategy, from its planning to its completion. It requires the debriefer to have expertise and training in the method, experience with simulation, andragogy and muldisciplinarity as well as with the clinical practice of care, among others^(^ 20 ^)^.

Some authors^(^ 8 ^,^ 11 ^,^ 15 ^,^ 21 ^)^ report that learning depends on reflection and integration of the experience. Reflection can be taught with availability and active engagement, under the orientation from an effective debriefer. The authors cited affirm that the debriefer‘s skills contribute to the best possible learning, highlighting that, without orientation, the student may inadvertently make mistakes and focus on negative attitudes.

As highlighted, the global analysis of the performance of the groups (Table 2) showed a disadvantage to group B (control), evidencing significance (p<0.001) and reinforcing the importance of the use of debriefing for learning. After the crossover, the students of this group improved their score in the third EDC. This probably occurred because they could participate in the scenarios with debriefing, that is, they could reflect on their practices, identify mistakes and fill gaps, which enabled a better performance in the evaluation. The results of this phase reinforced the evidence of the contribution of debriefing.

The items on the checklist of the second EDC, in which statistical significance was found (p<0.001) for the experimental group, were as follows: To schedule the vaccines that the child will receive next month in the vaccination card; to fill the vaccination record correctly; to record the vaccinations given on the map; to check the batch and the validity of the vaccine; to store the vaccine vials on the refrigerator; to advice the mother regarding the correct positioning of the child according to the vaccine to be administered (oral administration, vastus lateralis muscle), and to administer the vaccination in the correct area, using the correct technique.

Identifying such items was particularly important for the study, as it not only validates the teaching method, but also reiterates fundamental aspects for its recommendation in the nursing care in the vaccine room.

The advantages attributed to debriefing have pointed out this phase of the experiential learning process as the most important component of a learning experience, strongly agreeing with the literature for the recommendation that every simulation based on learning experience includes debriefing planning^(^ 8 ^,^ 17 ^,^ 19 ^,^ 21 ^-^ 23 ^)^.

The results highlight the importance of reflection on experiential learning, and debriefing is a valuable component for production and gain of knowledge. Thus, its use should be recommended as a strategy to facilitate the teaching and learning processes of nursing care.

Finally, the study could have been more effective with the possibility of measuring the students’ clinical performance in the actual practice, which was not possible. Therefore, studies that include not only the evaluation of the student in clinical practice after realistic scenarios with and without debriefing, but also the opinion of patients or clients regarding the care provided by the students in the nursing internships are recommended.

## Conclusion

The results of this investigation show the students of the experimental group showed the best learning outcomes when compared with the control group, which strengthened the evidence that the use of scenarios with debriefing was actually effective for learning, corroborating the hypothesis that this teaching technique improves the clinical performance in nursing care.
